# Correction to “A cross‐sectional study on perceived workplace health support and health‐related quality of life”

**DOI:** 10.1002/1348-9585.12395

**Published:** 2023-03-18

Kurogi K, Ikegami K, Eguchi H, et al. A cross‐sectional study on perceived workplace health support and health‐related quality of life. *J Occup Health*. 2021;63:e12302.

In Table 2, the description was incorrect. The Table 2 with correct description is given below.

**TABLE 2:** Comparison of the scores of the CDC HRQOL among groups according to perceived workplace health support (PWHS).

Parameters	Groups according to PWHS^a^	Sex‐age‐adjusted			Multivariate^b^		
		Odds ratio	95% CI^c^	*P*	Odds ratio	95% CI^c^	*P*
Self‐rated health^d^	Very high	0.15	[0.13–0.16]	<.01	0.15	[0.14–0.16]	<.01
	High	0.30	[0.28–0.32]	<.01	0.31	[0.29–0.33]	<.01
	Low	0.58	[0.54–0.63]	<.01	0.60	[0.56–0.64]	<.01
	Very low	Reference			Reference		
Physically unhealthy days (# days)	Very high	0.39	[0.36–0.43]	<.01	0.41	[0.37–0.45]	<.01
	High	0.44	[0.42–0.47]	<.01	0.46	[0.43–0.49]	<.01
	Low	0.64	[0.60–0.68]	<.01	0.67	[0.62–0.71]	<.01
	Very low	Reference			Reference		
Mentally unhealthy days (# days)	Very high	0.28	[0.25–0.31]	<.01	0.29	[0.26–0.32]	<.01
	High	0.34	[0.32–0.36]	<.01	0.35	[0.33–0.38]	<.01
	Low	0.54	[0.51–0.58]	<.01	0.56	[0.52–0.60]	<.01
	Very low	Reference			Reference		
Days with activity limitation (# days)	Very high	0.42	[0.38–0.47]	<.01	0.44	[0.40–0.49]	<.01
	High	0.45	[0.42–0.48]	<.01	0.46	[0.43–0.49]	<.01
	Low	0.65	[0.60–0.70]	<.01	0.67	[0.62–0.72]	<.01
	Very low	Reference			Reference		

^a^ PWHS: perceived workplace health support.

^b^ The multivariate model was adjusted for sex, age, educational background, presence of illnesses that require hospital treatment, employment status, company size, and working hours per day.

^c^ CI, confidence interval.

^d^ Self‐rated health (5‐point Likert scale): 1. Excellent 2. Very good 3. Good 4. Fair 5. Poor.

We apologize for this error.

